# Cognitive and affective reflection increases appreciation for less preferred subcategories of experiential goods

**DOI:** 10.3389/fpsyg.2023.1271516

**Published:** 2023-12-21

**Authors:** Cammy Crolic, Chris Janiszewski

**Affiliations:** ^1^Saïd Business School, University of Oxford, Oxford, United Kingdom; ^2^Warrington College of Business Administration, University of Florida, Gainesville, FL, United States

**Keywords:** negative attitude, attitude change, experiential consumption, aesthetic appreciation, perception, reflective appraisal

## Abstract

Attitudes, particularly negative attitudes toward experiential goods, are difficult to change. As a result, people tend to choose and consume experiential goods from their preferred subcategory (e.g., prefer impressionist art so primarily choose to view impressionist paintings) while disregarding options from less preferred subcategories (e.g., ignore cubist or surrealist paintings). This research investigates the consequences of reflection while consuming experiential goods from less preferred subcategories. Namely, an initial, negative reflexive response can be overridden by a reflective appraisal which increases appreciation for experiential goods from less preferred subcategories. Six studies show how a reflective appraisal differs from a reflexive response (i.e., a reflective appraisal has more cognitive and affective thoughts than evaluative thoughts, respectively), that reflective appraisals can supplant reflexive responses to experiential goods in less preferred subcategories, and that reflective appreciation training encourages reflective appraisal. A reflective appraisal improves the intent to consume, enhances appreciation of the consumption, and increases the consumption of novel experiential goods in less preferred subcategories.

## 1 Introduction

Attitudes, particularly negative attitudes toward experiential goods, are difficult to change. This is especially true for experiential goods (e.g., music, art, film, wine, and food), goods whose perceptual attributes and quality cannot be determined until they are experienced (Nelson, 1970). Experiential goods tend to be hedonic in nature in that they are consumed for the mere pleasure of the consumption experience (Alba and Williams, 2013). As a result, people tend to select experiential goods from their preferred subcategory (e.g., people who prefer impressionist art choose to view impressionist paintings) while disregarding options from less preferred subcategories (e.g., people who prefer impressionist art ignore cubist or surrealist paintings). For example, a lover of pinot grigio might repeatedly select a bottle of pinot grigio over a chardonnay, riesling, and sauvignon blanc. Similar behavior occurs for other experiential categories such as music (e.g., listening to rock music over pop, classical, and jazz), film (e.g., selecting action movies over romance, comedy, and drama), and food (e.g., preferring Thai food to French, Italian, and Mexican).

Subcategory loyalty has been attributed to several factors: the difficulty of changing attitudes, particularly negative attitudes (Rozin and Fallon, 1986), a desire to maximize utility and increase overall satisfaction (Savage, 1954), a desire to decrease the risk of a substandard outcome (Bettman, 1973), a desire to simplify decision-making (Hoyer, 1984; Iyengar and Lepper, 2000), and routine or habit (Hoyer, 1984; Murray and Häubl, 2007).

In business, a significant amount of effort is devoted to expanding the number of subcategories that individuals will consider and consume. People tend to buy and consume more, when there are more variants in a product line (Slotegraaf and Pauwels, 2008; Aurier and Mejía, 2020) or when more subcategories surpass an acceptability threshold (Brynjolfsson et al., 2003). Thus, there are numerous programs aimed at helping people learn to consider a wider variety of experiential goods from different subcategories. For example, the wine industry sponsors wine-tasting events, educates people about pairing different varietals of wine with different types of food, and encourages retailers to offer mixed-case discounts. Similarly, retailers offer beer, cheese, chocolate, olive oil, vinegar, wine, and whiskey tasting seminars. These activities are designed to encourage attitude change which would result in people being open to considering and consuming a wider variety of experiential goods.

There is one potential drawback to the effort to increase the range of experiential goods that people select and consume in a product category—it is only successful to the extent that people learn to better appreciate, and actually enjoy, goods in less preferred subcategories (i.e., value the consumption experience). To illustrate, consider a wine distributor who offers consumers incentives to purchase wine in less preferred subcategories (e.g., the pinot grigio segment is encouraged to buy bottles of chardonnay and sauvignon blanc). If a purchase occurs, but there is insufficient appreciation for the options in the less preferred subcategories, there could be negative consequences. First, a stockpile of less preferred options could discourage future purchases in the category (e.g., “I don't need to buy more wine. I need to drink what I already have”) (Mela et al., 1998). Second, to the extent a person engages in the consumption of experiential goods from a less preferred subcategory, and the person has limited appreciation for the experience, dissatisfaction may generalize to the entire category and discourage future purchases (e.g., “Drinking wine is not as satisfying as it used to be.”) (Botti and Iyengar, 2004).

The foregoing discussion illustrates the importance of identifying appropriate strategies for encouraging attitude change by enhancing the appreciation of less preferred experiences (Carbone and Haeckel, 1994; Ochsner and Gross, 2005; Kashdan et al., 2015). We propose a strategy that relies on the fact that people have two approaches to valuing an experience (Arnold, 1960a; Lieberman, 2003; Kappas, 2006; Frijda, 2009; Strack and Deutsch, 2014). First, there is a reflexive response that is resistant to change (Wilson et al., 2000; Frijda, 2010; Howe and Krosnick, 2017). A reflexive response is an immediate positive/negative attitude that is strong (Fazio et al., 1984; Petty and Krosnick, 1995; Ajzen, 2001), stable over time (Howe and Krosnick, 2017), and influential on behavior (Krosnick and Petty, 1995; Ajzen and Fishbein, 2000; Ajzen et al., 2018). Second, there is a reflective appraisal that allows for attitude change (Petty and Brinol, 2010; Bohner and Dickel, 2011). Reflection is associated with introspection and reasoned judgment (Kember et al., 1999; Frijda, 2009; Strack and Deutsch, 2014). A reflective appraisal is more controlled, more affectively nuanced, and more personal (i.e., reflects how the experience relates to the consumer) (Lieberman, 2003; Kappas, 2006; Frijda, 2009). Reflective appraisals can rely on the same sensory information as the reflexive response, yet they do not always occur (Evans and Stanovich, 2013). We propose that encouraging a person to utilize a reflective process, during the consumption of an experiential good from a less preferred subcategory, provides an opportunity to generate favorable reflective appraisals that can supplant the automatic, negative reflexive response. Furthermore, to the extent these reflective appraisals are practiced, they may be able to modify future negative, reflexive responses (e.g., learning to appreciate one chardonnay increases appreciation for all chardonnays).

This research makes four important contributions. First, it provides additional insight into the ability to change negative attitudes (Petty and Brinol, 2010). Second, it provides a new strategy for increasing the appreciation of less preferred experiential goods (i.e., altering an initial, reflexive response). Prior research has emphasized mere exposure (Mantonakis et al., 2008), expectation framing (Deliza and MacFie, 1996; Spence and Ngo, 2012), persuasion (Hamari et al., 2014), deliberation (Fazio, 1990), and habit change (Hermsen et al., 2016) as approaches to increasing the appreciation of less preferred experiential goods. We show that people can be encouraged to alter the way they respond to the sensory information provided by a consumption experience. Third, prior approaches to changing appreciation tend to be product-specific (e.g., deliberation focuses on attitude change only of the attitude object; Fazio, 1990). In contrast, our reflection process should generalize to experiential goods in other subcategories (e.g., learning to appreciate a previously disliked chardonnay makes it easier to appreciate a previously disliked pinot noir). Finally, our findings show that reflection during a consumption experience can be beneficial. Prior research has primarily shown that reflection reduces appreciation for a favorite experiential good (Wilson and Schooler, 1991; Halberstadt, 2010). Contrarily, we show that reflection can alter attitudes and increase appreciation for a less preferred experiential good.

## 2 Theoretical background

### 2.1 Responses to consumption of experiential goods

Dual-process models assume behavior is guided by two constellations of processes: (1) non-deliberate, reflexive, associative, affective-based processes and (2) deliberate, reflective, analytic, rule-based processes (Evans, 2008; Evans and Stanovich, 2013). The dichotomy of processes emphasized in a dual-process investigation depends on the domain (e.g., experience, learning, and decision-making). Dual-process models investigating experience coalesce around the reflexive–reflective process dichotomy. Arnold (1960a,b) proposes that a response to a consumption experience can be direct, immediate, and intuitive, in the sense that it is good or bad. Alternatively, the appraisal of an experience can be a reflective modification of the initial response, in that it is more reasoned, meaningful, and personally relevant. Frijda (2009, 2010) proposes that there are both an impulsive response that is reactive, urgent, and action-oriented and a reflective appraisal that incorporates additional information to generate a more deliberate, goal-directed response. Lieberman (2003) proposes there is a reflexive response, which is an immediate evaluation, and the potential for a reflective appraisal, which should occur when the initial response feels inappropriate. Common to these models is a reflexive, evaluative response that supports an action tendency and a reasoned, deliberate reflective appraisal that modifies the reflexive response and/or generates a novel response. Building on these dual-process models, we show that appreciation training is effective at encouraging consumers to use their reflective appraisals which can change attitudes (i.e., liking) and behavior (e.g., intention and amount) for less preferred experiential goods.

#### 2.1.1 Reflexive responses

A reflexive response is an immediate reaction to a consumption experience. These immediate responses typically consist of a positive or negative evaluation of the experience (i.e., attitudes) (Arnold, 1960a,b; Pham et al., 2001; Ferguson and Bargh, 2008; Frijda, 2009). Although these responses can be innate, a large majority of these responses are over-learned and automatic (Fazio et al., 1986; Wilson et al., 2000; Evans and Stanovich, 2013). For example, people may initially prefer tea to coffee because it is less bitter and more floral but eventually simply class tea as “good tasting” and coffee as “bad tasting.” These potentially automatic associations between perceptions and responses (positive and negative evaluations) derive from a history of consumption experiences (Ferguson and Bargh, 2008; van Osselaer and Janiszewski, 2012). Functionally, they facilitate an immediate positive/negative attitude to an experiential good (Allport, 1935; Fazio et al., 1986; Wilson et al., 2000). The implication is that the response to many experiential goods, including food, art, and music, is determined, in part, by prior experiences in the category (Kolb, 1984; Schmitt, 1999, 2010; Lakshmanan et al., 2010).

#### 2.1.2 Reflective appraisals

A reflective appraisal relies on the same sensory input that supports a reflexive response, but it can incorporate affective and cognitive associations beyond those provoking the immediate reflexive response. Reflective appraisals are more deliberate (Lieberman, 2003; Frijda, 2009), more affectively nuanced (Frijda, 2009), more cognitively reasoned (Lieberman, 2003), more self-referential (Arnold, 1960a,b), and less judgmental (Garland et al., 2015). Increasing the amount of thought generated in response to a consumption experience allows a person to combine and/or replace an immediate evaluative response (e.g., positive/negative attitude) with more reflective cognitive (e.g., experiential characteristics, benefits, and usage context) and affective (e.g., feelings, self-referential thoughts, and interest/engagement) thoughts (Kember et al., 1999; Strack and Deutsch, 2014; Garland et al., 2015). Reflective appraisals allow for the consideration of more inputs than simple, immediate perceptions and, hence, tend to require more conscious thought (Lieberman, 2003). Reflective appraisals also provide an opportunity to create novel thoughts and, therefore, are instrumental to how people expand their domain of preferences (Holbrook and Hirschman, 1982; Gregan-Paxton and John, 1997; Birch, 1999; Kolb et al., 2001). That is, reflective appraisals can help reinforce and/or create associations between sensory information and the appreciation of this information through cognitive and affective processes.

### 2.2 Learning to appreciate less preferred experiential goods

Reflection provides an opportunity to alter negative, reflexive responses to a less preferred consumption experience. Yet, it is not sufficient to simply direct people to be reflective and hope that overrides negative attitudes. Instead, people need to be encouraged to access the affective and cognitive components of their consumption knowledge base and use them in a way that can lead to a more enjoyable consumption experience. To the extent the processes that lead to more positive reflective appraisals can be practiced, and reinforced, they can start to co-occur with, or replace, existing negative reflexive responses (Wilson et al., 2000). We refer to this strategy of encouraging access to their consumption knowledge base as *appreciation training*.

We propose that appreciation training can enhance consumption experiences via co-occurrence or replacement. There are two co-occurrence strategies. First, people can be encouraged to associate a positive outcome with a novel, sensory characteristic of a consumption experience (Warlop et al., 2005). For example, a pinot grigio lover may be taught to identify the buttery notes in a chardonnay (a novel, sensory characteristic) and to associate a positive outcome with this flavor profile (e.g., the wine is “fresh”). Second, people can be encouraged to associate a positive outcome with a known, sensory characteristic of a consumption experience (Hoegg and Alba, 2007; Spence et al., 2010). For example, a pinot grigio lover may know chardonnays are oaky but need to be taught that oakiness suggests longer aging and higher quality. Appreciation training also enhances consumption experiences through a replacement strategy. People can be encouraged to replace a negative association with a positive one (Faerber et al., 2010). For example, a pinot grigio lover may experience chardonnays as light, which they may automatically interpret as flavorless, but appreciation training might alter the interpretation of light to mean delicate and sophisticated.

**Figure 1:**
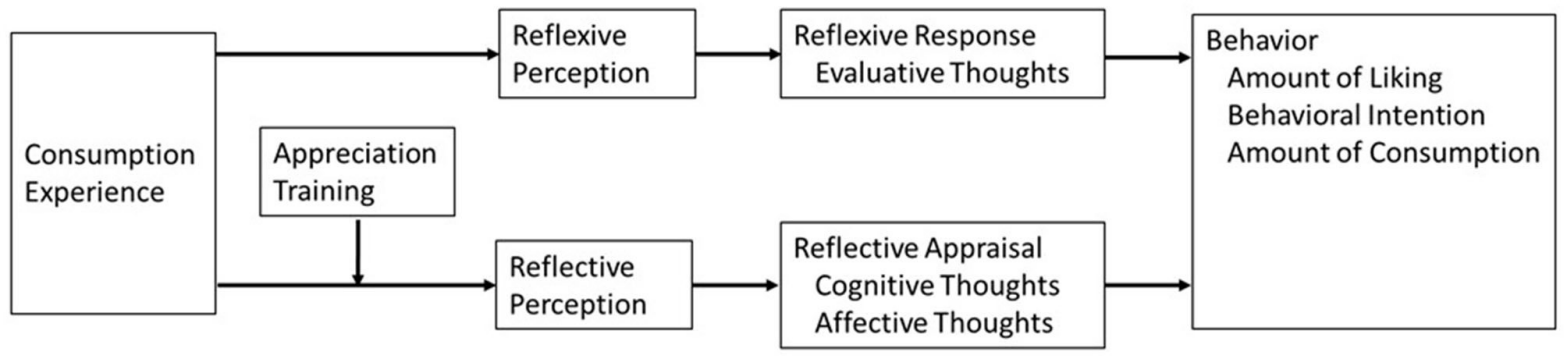
Model.

These ideas are summarized in the model presented in Figure 1. The model posits that people have a reflexive perception of the sensory components of a consumption experience (e.g., perceive the light flavor of a chardonnay) and then have a reflexive evaluative response (e.g., light flavor is bad), resulting in a negative attitude toward the wine and lower subsequent consumption behaviors. Yet, the reflexive response can be overwritten, amended, or supplanted with appreciation training. Appreciation training encourages a person to be more reflective during a consumption experience, which may result in either the same or different (reflective) perceptions of the sensory experience (e.g., perceive the buttery flavor of a chardonnay in addition to the light flavor). Reflection results in cognitive and affective thoughts about the consumption experience (e.g., buttery flavors mean the wine is fresh, and the light flavor is sophisticated which makes me feel content). The cognitive and affective thoughts comprise a reflective appraisal that is in addition to, or an alternative to, the reflexive response (Wilson et al., 2000), resulting in attitude change and higher subsequent consumption behaviors. Finally, we propose that appreciation training will be more effective when it is conducted in the less preferred subcategory as less preferred subcategory experiences generate negative responses that can change because of training.

There is indirect evidence that is consistent with the ideas summarized in Figure 1. First, “difficult to solve” art is disliked. An information card or docent who helps “solve the art” via reflection provides meaning, understanding, and aesthetic value (Muth et al., 2015). This suggests that when reflective appraisals are difficult to generate, people tend to rely on their negative reflexive response. Yet, a forced reflective appraisal can improve consumption experiences and encourage more favorable attitudes. Second, negative responses to certain genres of music are immediate. Encouraging listeners to develop a more complete framework for analyzing music, a reflective process, can result in associations between features in disliked music genres and benefits in liked music genres (Huang, 1997). Finally, in the domain of food consumption, training to eat mindfully (e.g., deliberately see, feel, taste, and smell raisins while eating them) increased the evaluation of unsampled, disliked foods (e.g., anchovies, Brussels sprouts, and sardines) but not unsampled, liked foods (e.g., pork, rice, and fig newtons) (Hong et al., 2011).

## 3 Research outline

The appreciation training hypothesis was tested using six studies. Study 1 demonstrated that reflective appreciation training (i.e., training that encouraged a person to experience an experiential good in a new way) resulted in positive attitude change, with a greater appreciation for experiential goods from less preferred subcategories. Study 2 provided process evidence; it showed that appreciation training encouraging reflection on experiential goods from less preferred subcategories resulted in more positively valenced cognitive and affective responses when later consuming experiential goods from less preferred subcategories. In turn, these responses increased the appreciation for novel goods in less preferred subcategories. Study 3 manipulated the proposed process by showing that reflective processing, a hypothesized consequence of the appreciation training encouraging reflection, increased the appreciation of the experiential goods in the less preferred subcategories. Study 4 demonstrated that appreciation training encouraging reflection influenced the liking of (4a), intent to consume (4b), and consumption of (4c) novel experiential goods in less preferred subcategories. This research has been conducted in accordance with guidelines on study ethics. All measures, manipulations, and exclusions in the study are disclosed. Sample size was determined by prevailing norms at the time of data collection; earlier studies aimed for at least 50 participants per cell and studies run later used power analysis to determine reasonable sample sizes to achieve the desired power. All data, analysis code, and research materials are available in the OSF repository: https://osf.io/e8wh4/?view_only=36ddc730e9bb4dd084ee0b8b003c29b1. Where applicable, preregistration is noted.

## 4 Study 1

We hypothesize that appreciation training can change people's attitudes toward a consumption experience (see Figure 1). As discussed earlier, reflexive responses should more directly correspond to evaluative judgment (Fazio et al., 1986; Wilson et al., 2000; Pham et al., 2001). Reflective appraisals should be more cognitive and affectively nuanced (Arnold, 1960a; Lieberman, 2003; Frijda, 2009). Thus, we anticipated that encouraging a person to reflect when judging a consumption experience from a less preferred subcategory should provide an opportunity to consider and/or develop new, favorable associations between perceptions and cognitive thoughts and/or perceptions and affective thoughts about the experience. Consequently, there should be an increased enjoyment of subsequent consumption experiences in the subcategory (see Figure 1).

Study 1 used music as the experiential category. Responses to music can be reflexive and reflective (Nieminen et al., 2011). First, there is an immediate, evaluative response that is resistant to change and results in a positive/negative attitude (Wilson et al., 2000; Frijda, 2010; Howe and Krosnick, 2017). Second, there can be a more nuanced reflective appraisal that depends on memories, associations, reflections, and affective judgments (Hargreaves and Colman, 1991; Sloboda, 1992; Woody and Burns, 2001; Robinson, 2009; Nieminen et al., 2011; Brattico et al., 2013). The reflective appraisal can modify and/or supplant the initial evaluative response (Leder et al., 2004; Reber et al., 2004; Brattico et al., 2013) and result in attitude change (Bohner and Dickel, 2011). Music from a favorite subcategory tends to encourage both responses, whereas music from less preferred subcategories tends to encourage only the initial reflexive response (Price and Swanson, 1990). Consequently, using music as our experiential good allowed us to test whether appreciation training can increase the appreciation of music from less preferred subcategories due to the cognitive and affective thoughts that emerge from reflection.

### 4.1 Method

#### 4.1.1 Design and participants

Three hundred twenty-five Amazon Mechanical Turk (MTurk) workers (median age = 33, 44.9% female workers, median education = bachelor's degree) participated in return for a payment. This sample size allowed us to detect an effect size of Cohen's *d* = 0.24 with 0.80 power. Participants were randomly assigned to one of two conditions (appreciation training: none—reflexive, reflective) in a between-subject design. The dependent measures were the type of thoughts generated and the liking of the experience.

#### 4.1.2 Procedure

First, participants ranked four styles of music: classical, pop, hip-hop, and rock. To assist participants in this process, each style was illustrated using a short song clip from the style. Participants were reminded to rank the entire style of music, not the sample song clip.

Next, the participants in the reflective condition received appreciation training instructions. Participants were told that there was a new way to listen to music. They were asked to listen to a sample song using this new approach. Specifically, they were told “Imagine this song was background music in the movie about your life. What would you be doing while this music was playing?” This manipulation was based on Arnold's (1960a,b) position that reflective appraisals are more self-relevant. Participants in the control condition (i.e., reflexive condition) were not given any special instructions. All participants then listened to a sample song in their third-ranked style of music (i.e., less preferred subcategory) for 30 s.

After listening to the sample song, participants listened to a novel target song in their third-ranked style for 1 min. The reason for this procedure is we wanted to assess whether the influence of the experimental manipulation on the sample song (i.e., encouraging a person to be more reflective) influenced the appreciation of a target song from a less preferred subcategory. After listening to the target song, participants were then presented with 30 words: 10 evaluative words (i.e., like, average, enjoy, good, excellent, appreciate, awful, bad, dislike, and despise), 10 cognitive words (i.e., beat, tempo, texture, melody, tune, rhythm, pitch, chorus, vocals, and instrumentals), and 10 affective words (i.e., happy, excitement, nostalgia, romantic love, calmness, joy, sad, anger, pain, and anxiety) in random order. They were asked to select between two and five words that best described what was going through their mind while listening to the song. Then, participants rated how much they liked the song (1 = not at all, 7 = like it a lot). Afterward, they answered demographic questions.

### 4.2 Results

#### 4.2.1 Liking analysis

Appreciation training increased liking of the less preferred song compared to no training [*M*_notraining =_ 4.09, SD = 1.81, *M*_training_ = 4.48, SD = 1.58; *t*_(323)_ = 2.07, *p* = 0.039, *d* = 0.23].

#### 4.2.2 Thought analysis

The number of cognitive and affective words was calculated as a proportion of the total words selected. Appreciation training increased the proportion of cognitive and affective thoughts compared to no training [*M*_notraining_ = 0.68, SD = 0.31, *M*_training =_ 0.77, SD = 0.27; *t*_(323)_ = 2.72, *p* = 0.007, *d* = 0.30].

#### 4.2.3 Mediation analysis

A mediation analysis (Hayes, 2013; process model 4) was used to assess whether cognitive and affective responses could account for the influence of the training on the liking of the song from the less preferred subcategory. As predicted, the proportion of cognitive and affective thoughts mediated the influence of training on liking of the song from the less preferred subcategory (β = 0.038, SE = 0.024, CI = 0.002 to 0.094).

### 4.3 Discussion

The results of study 1 show that an appreciation training program that focuses on encouraging reflection (vs. no training where participants presumably rely on their initial reflexive responses) can change negative attitudes by increasing the liking of an experiential good from a less preferred subcategory. Appreciation training changed the way people interpreted the experience which, in turn, influenced their liking of the experiential good. This study also provides initial evidence for reflection by demonstrating the mediating influence of cognitive and affective thoughts on the liking of the experiential good.

## 5 Study 2

Study 1 established that appreciation training can increase the enjoyment of an experiential good from a less preferred subcategory. Our claim is that appreciation training encourages a person to reflect, as opposed to relying on a reflexive response, when experiencing goods from less preferred subcategories. We further claim that reflection creates new associations (i.e., new ways of appreciating consumption experiences) that generalize to other experiential goods in the less preferred subcategory. These new reflective appraisals can result in attitude change (Bohner and Dickel, 2011). Yet, the training in study 1 could have simply encouraged people to have positive thoughts which increased the enjoyment of any consumption experience. If this is true, any training program could encourage positive thoughts, so that a no training control group is, by definition, an inappropriate control group. To address this issue, study 2 used appreciation training that encouraged reflective or reflexive processing.

Study 2 also measured thoughts but in a more nuanced way than study 1. Participants were asked to provide their thoughts as they listened to each song. These thoughts were coded by two research assistants who were blind to the intent of the study (*r* = 0.70). Disputes were resolved by the second author, who was blind to the condition. Each response was coded for its category (e.g., evaluative, cognitive, affective, and other) and valence (positive, neutral, negative), which allowed us to code the valence of the thoughts in each response category. We expected that the averaged valence of evaluative thoughts (an indicator of a reflexive response) and the averaged valence of cognitive/affective thoughts (an indicator of a reflective appraisal) would mediate the influence of reflective appreciation training on enjoyment of the target songs from the less preferred subcategories (see Figure 1). This is because reflective appreciation training can change initial attitudes by encouraging a person to engage in reflection that generates thoughts in addition to the reflexive, evaluative thoughts that occur automatically and form the initial attitude. We did not expect an influence of reflective training on the enjoyment of songs from the preferred subcategory because these songs should be liked and, hence, not benefit from reflection (i.e., both reflexive and reflective training would yield a “good” response, either a positive evaluation or positive cognitions and affect).

### 5.1 Method

#### 5.1.1 Design and participants

Two hundred seventy-one MTurkers (median age = 33, 44.6% female workers, median education = some college) were randomly assigned to one of three conditions (training frame: no training control group, reflexive conventional frame, and reflective unconventional frame) in a between-subject design with a repeated-measure dependent variable (song liking) as a within-subject factor. Sample size allowed us to detect an interaction with Cohen's *f* effect size of 0.08 with 0.80 power. There were four repeated measures: the liking of a song from the first-, second-, third-, and fourth-ranked subcategories. A planned contrast of these repeated measures, the liking of the song from the favorite subcategory compared to the average liking of the songs from the three less preferred subcategories, was a within-subject factor. Four participants were removed from the analysis for failing to properly complete the training (e.g., answered non-sensically, suggesting they failed to attend to the instructions or that they were a bot), leaving a final sample of 267 people.

#### 5.1.2 Procedure

First, participants ranked four styles of music: classical, pop, hip-hop, and rock. To assist participants in this process, each style was illustrated using a short song clip from the style. Participants were reminded to rank the entire style of music, not the sample song clip.

Next, the participants in the training conditions received the training frame instructions. Participants in the reflexive conventional frame condition were told “The training you are about to go through is standard training. It will provide an established, traditional training to appreciate music.” Given this was status quo training, we expected it would encourage participants to respond to music as they usually would, reflexively. Participants in the reflective unconventional frame condition were told “The training you are about to go through is novel training. It will provide a unique, alternate training to appreciate music.” Given this novel training, we expected it would encourage participants to process the music more carefully than they usually did (i.e., reflectively).

A manipulation check of these frames, using 86 MTurk participants from outside the main study, exposed participants to the experimental procedure up to the point of the actual training. They were then asked the degree to which they expected the training would be reflective: “make me spend more time thinking about the music,” “make me reflect more on the music,” “encourage me to think more deeply about the music,” “make me spend less time thinking about the music,” “make me make a snap judgment about the music,” and “encourage me to think superficially about the music” (the latter three items were reverse-coded; α = 0.70). The results showed the reflective training frame was perceived as more reflective than the reflexive training frame [*M*_reflective_ = 5.25, SD = 0.87, M_reflexive_ = 4.81, SD = 1.06; *t*_(84)_ = 2.10, *p* = 0.038, *d* = 0.45].

Participants in the two training conditions were then trained in three components: tempo, melody, and texture. The training materials were developed using materials from a music appreciation course. Each component of training used a song from one of the three less preferred subcategories. For example, if a participant ranked rock music as their favorite, they were trained on tempo in hip-hop, melody in pop, and texture in classical. Training in each of the three components involved the following: (1) reading instructional information, (2) trying to apply the instructional technique to the accompanying song (same song as used as example clips in the ranking task to reduce additional exposure to the music style), and (3) reading feedback about the application of the technique to the same song (see Table 1 for an example of training when the favorite subcategory is rock). For example, training on tempo in hip-hop said:

Music has tempo, or the speed or pace of the song. Traditionally, this is measured in beats per minute. To illustrate, consider the Hip Hop song below. The artist chooses the tempo to set the mood of the piece, convey energy, and deliberately change the tempo to capture your attention. Please take a moment to listen to the song and reflect upon how its tempo makes it appealing.

**Table 1:** Study 2 music training.

**Training for people whose favorite subcategory is rock**			
	**Instructional information**	**Application**	**Application feedback**
Step 1: TempoHip-Hop	• Music has tempo, or the speed or pace of the song. Traditionally, this is measured as beats per minute. To illustrate, consider the hip-hop song below. The artist chooses the tempo to set the mood of the piece, convey energy, and deliberately change the tempo to capture your attention. Please take a moment to listen to the song and reflect upon how its tempo makes it appealing • [Clip of All I Think About is You by Nelly featuring Kelly Rowland]	Can you provide a comment about the tempo? Do not worry about coming up with a “correct” response, just focus on trying	For example, the song below uses tempo to set a sultry mood to the music and convey passionate emotions. The tempo drastically changes 23 s into the song as the energy picks up. Eventually, the artist begins incorporating rap to reinforce his fast-talking, confident approach to the whirlwind romance. The tempo helps reinforce the mood, energy, and lyrics
Step 2: MelodyPop	• Songs can have quality melodies and are what is commonly referred to as “the tune.” A melody is the combination of rhythm (the length of notes) and pitch (the frequency of the notes—or how high or low they sound). Pop music is enjoyable because the melody is repetitive, has prominent vocals, uses familiar melody patterns, and mixes in high and low notes • [Clip of Perfect by Ed Sheeran]	Can you tell us something about the melody? Again, do not worry about coming up with a “correct” response	For example, this pop song uses a repetitive melody, which is catchy and easy to learn. The song also has strong, clear vocals. Additionally, the song uses a familiar melody closely related to the sound of other songs that allows it to sound familiar (in a good way, like a favorite hoodie) while incorporating fresh, new elements
Step 3: TextureClassical	• The texture of the song is the way in which different lines of music interweave. It is the overall quality of the sound in a song. Classical music has a light, clear texture and is less complex, usually opening lightly and becoming thicker. It is mainly homophonic, which means using a clear melody line over an accompaniment • [Clip of Four Seasons Autumn by Vivaldi]	Can you identify something about the texture of the song?	*For example, this song is a bit unique in that it opens with a thick texture and then thins out abruptly. In this song, the texture alternates between thin and thick. Also, the quality of the song is crisp and clear. As with typical classical music, you can clearly identify the melody and the accompaniment*

Participants then listened to a hip-hop song and were asked, “Can you provide a comment about the tempo? Do not worry about coming up with a ‘correct' response, just focus on trying.” After responding, participants were given feedback about the specifics of the tempo of the song. Note that the training focused on how to appreciate music, as opposed to providing factual knowledge about music history, music classification, music production, or music marketing.

After completing the training, or in the case of the no training control condition, after ranking the music styles, participants were told that they were going to listen to four new songs. In random order, participants were presented with a new song from each of the four styles. They listened to the music and were asked to describe what they were experiencing as they were listening to the song. Then, participants were asked to rate how much they liked the song (1 = not at all, 7 = like it a lot). Afterward, they answered questions about their expertise, the credibility of the training (only in the two training conditions), intrinsic motivation, and demographics.

### 5.2 Results

#### 5.2.1 Data preparation

There were 24 possible ranking orders of the four musical styles, so training material by training frame by music style analysis could not be performed. There was no training frame by preference rank (second, third, and fourth) interaction [*F*_(4, 528)_ = 2.12, *p* = 0.077], so the liking scores for the songs from the three less preferred subcategories were averaged. The liking score of the song from the favorite subcategory was the other measure in the within-subject factor.

#### 5.2.2 Liking analysis

**Figure 2:**
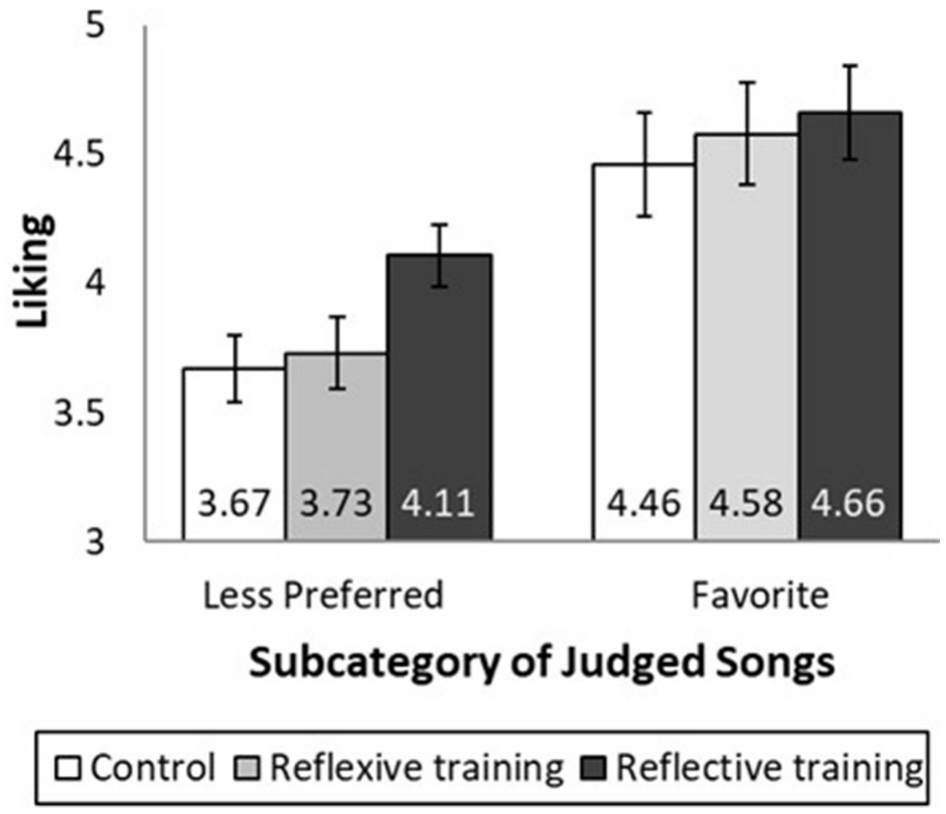
Liking of songs by type of appreciation training and subcategory. Participants received no training (control), reflexive training, or reflective training (between-subject factor). Participants rated a novel song from their most preferred subcategory (labeled “Favorite”) and novel songs from their second-, third-, and fourth-ranked subcategories (average of these ratings is labeled “Less Preferred”). Reflective training only increases the appreciation of songs in the less preferred subcategories. Error bars are ±1 SE.

The means are shown in Figure 2. Two analyses were performed: one to address the concern that appreciation training just creates more positive thoughts that would benefit disliked and liked subcategories equally and one to address the issue that a no training control group is inappropriate. With respect to the first analysis, we predicted that a comparison of the reflective training vs. the no training control group would show greater liking of the songs from the less preferred subcategories but not the song from the favorite subcategory. Consistent with this prediction, the training frame by subcategory interaction was significant [*F*_(1, 264)_ = 4.13, *p* = 0.043, *f* = 0.11]. Reflective training increased the liking of the songs in the less preferred subcategories [*M*_control_ = 3.67, SD = 1.23, *M*_reflective_ = 4.11, SD = 1.16; *F*_(1, 264)_ = 5.38, *p* = 0.021, *f* = 0.13] but not in the favorite subcategory [*M*_control_ = 4.46, SD = 1.93, *M*_reflective_ = 4.66, SD = 1.69; *F*_(1, 264)_ = 0.54, *p* = 0.464]. We also predicted that a comparison of the reflexive training vs. the no training control group would show no influence on any subcategory of songs. Consistent with this prediction, reflexive training did not increase the liking of the songs in the less preferred subcategories [*M*_control_ = 3.67, SD = 1.23, *M*_reflexive_ = 3.73, SD = 1.33; *F*_(1, 264)_ = 0.08, *p* = 0.778] or favorite subcategory [*M*_control_ = 4.46, SD = 1.93, *M*_reflexive_ = 4.58, SD = 1.92; *F*_(1, 264)_ = 0.22, *p* = 0.642]. Furthermore, the training by subcategory interaction [*F*_(1, 264)_ = 0.00, *p* = 0.957] was not significant.

In the second analysis, the reflective and reflexive training conditions were compared. Consistent with predictions, the training by subcategory interaction was significant [*F*_(1, 264)_ = 3.89, *p* = 0.050, *f* = 0.10]. Reflective training increased liking of the songs in the less preferred subcategories [*M*_reflexive_ = 3.73, SD = 1.33, *M*_reflective_ = 4.11, SD = 1.16; *F*_(1, 264)_ = 4.13, *p* = 0.043, *f* = 0.11] but not in the favorite subcategory [*M*_reflexive_ = 4.58, SD = 1.92, *M*_reflective_ = 4.66, SD = 1.69; *F*_(1, 264)_ = 0.07, *p* = 0.788].

#### 5.2.3 Mediation analysis

**Figure 3:**
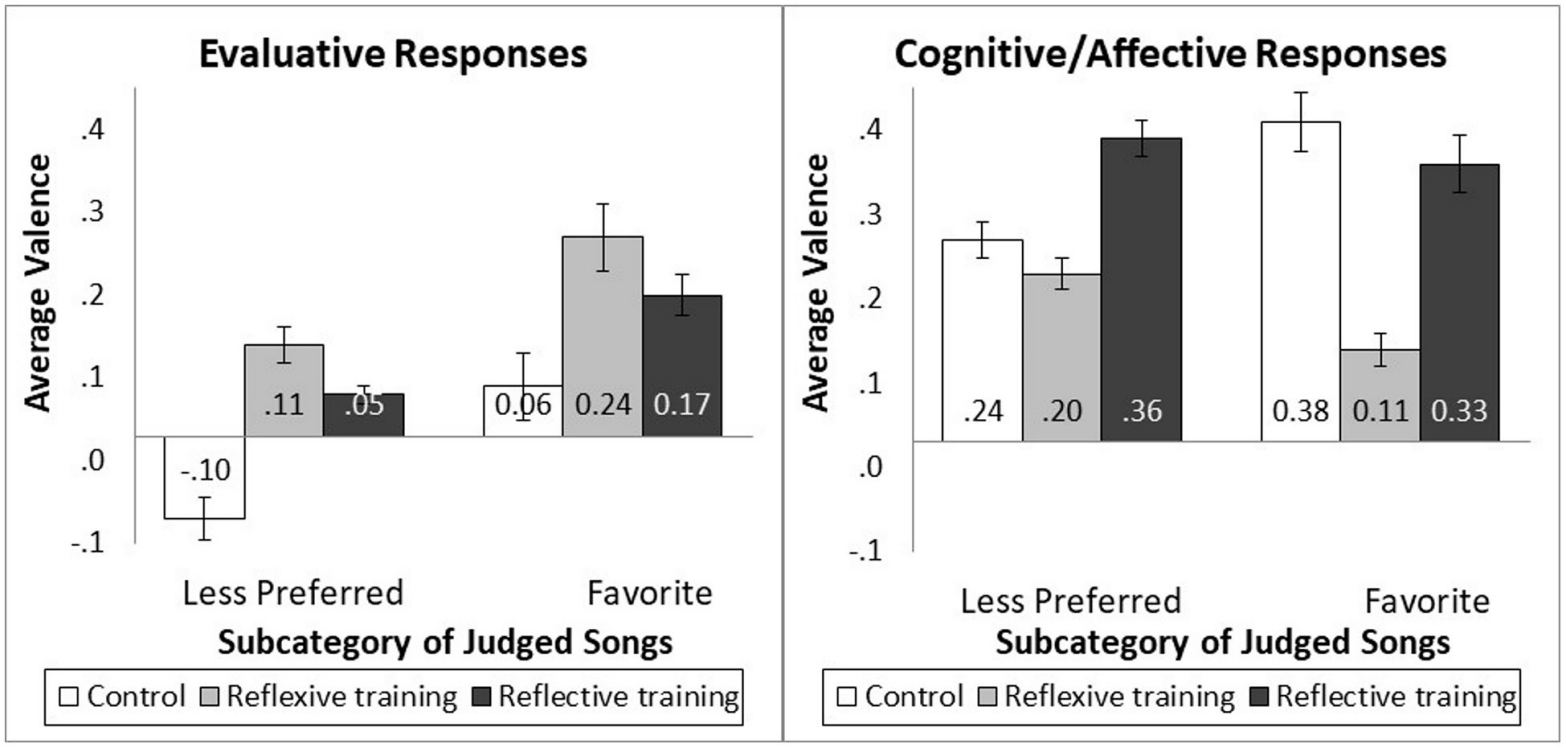
Evaluative and cognitive/affective response by training frame and subcategory.

We predicted both the valenced evaluative thoughts (reflexive) and the valenced cognitive/affective thoughts (reflective) should mediate the difference in appreciation for songs in the less preferred subcategories. The mediating variables were derived from participant descriptions of their listening experience for each song. First, the responses were coded into the categories of (1) purely descriptive/unrelated, (2) evaluative (e.g., “I like it,” “it's catchy,” and “I am enjoying this music”), or (3) cognitive/affective (e.g., “This music is relaxing,” “I feel like dancing,” and “I feel a sense of nostalgia and power”). Second, the evaluative and cognitive/affective responses were assigned a positive (“I like the beat,” “it makes me feel happy”), negative (“this is awful,” “I am feeling anxious”), or neutral valence. The valence of the responses in each category was then averaged, so that a score could range from −1 (i.e., all thoughts in the coded category were negative) to +1 (i.e., all thoughts in the coded category were positive). Consequently, each participant had four scores: an average evaluative score and an average cognitive/affective score for both the less preferred subcategory songs and the favorite subcategory song. The means for the less preferred subcategory songs and favorite songs are shown in Figure 3.

First, consider the reflective training by control group contrast. For evaluative responses, the training by subcategory interaction was not significant [*F*_(1, 264)_ = 0.16, *p* = 0.690], but the reflective training did increase the positivity of the evaluative responses [*M*_control_ = −0.02, SD = 0.42, *M*_reflective_ = 0.11, SD = 0.23; *F*_(1, 264)_ = 6.28, *p* = 0.013, *f* = 0.14]. For cognitive/affective responses, the training by subcategory interaction was significant [*F*_(1, 264)_ = 4.53, *p* = 0.034, *f* = 0.12]. Reflective increased cognitive/affective responses to songs in the less preferred subcategory [*M*_control_ = 0.24, SD = 0.34, *M*_reflective_ = 0.36, SD = 0.36; *F*_(1, 264)_ = 5.34, *p* = 0.022, *f* = 0.13] but not the favorite subcategory [*M*_control_ = 0.38, SD = 0.57, *M*_reflective_ = 0.33, SD = 0.56; *F*_(1, 264)_ = 0.42, *p* = 0.519]. The mediation analysis for the songs in the less preferred subcategories showed evaluative responses (β = 0.226, SE = 0.081, CI = 0.084 to 0.407) and cognitive/affective responses (β = 0.147, SE = 0.073, CI = 0.026 to 0.317) mediated the relationship between training (control vs. reflective) and liking. This finding is consistent with predictions.

Second, consider the reflective training by reflexive training group contrast. For evaluative responses, the training by subcategory interaction [*F*_(1, 264)_ = 0.01, *p* = 0.918] and training main effect (*M*_reflexive =_ 0.17, SD = 0.39, *M*_reflective_ = 0.11, SD = 0.23; *F*_(1, 264)_ = 1.28, *p* = 0.259] were not significant. For cognitive/affective responses, the training by subcategory interaction was not significant [*F*_(1, 264)_ = 0.46, *p* = 0.50], but reflective training did increase cognitive/affective responses to songs relative to reflexive training [*M*_reflexive_ = 0.15, SD = 0.24, *M*_reflective_ = 0.35, SD = 0.38; *F*_(1, 264)_ = 14.22, *p* < 0.001, *f* = 0.22]. The mediation analysis for the songs in the less preferred subcategories did not show mediation of evaluative responses (β = −0.056, SE = 0.051, CI = −0.180 to 0.027) but did show cognitive/affective responses (β = 0.164, SE = 0.076, CI = 0.048 to 0.351) mediated the relationship between training (reflexive vs. reflective) and liking. This finding is partially consistent with predictions.

### 5.3 Discussion

Study 2 demonstrated that appreciation training encouraging reflection can change negative attitudes by increasing the liking of experiential goods (e.g., songs) from less preferred subcategories. We attribute this increase in liking to reflection. Evidence for reflection is provided by the mediation analysis; when the appreciation training was reflective, both evaluative thoughts (indicators of a reflexive response) and cognitive/affective thoughts (indicators of reflection) were responsible for the increase in liking relative to a control group and the reflexive training group.

The results of study 2 are inconsistent with three alternative explanations. First, it is unlikely that reflective appreciation training simply encourages a person to be more engaged in the music, to have more positive thoughts, or to be more positively predisposed toward all music. If this had been the case, the liking of the song in the favorite subcategory would have increased. Second, it is unlikely that appreciation training encouraging reflection created familiarity, expectation, or persuasion. If this was the case, the liking of the song in the favorite subcategory should have increased. Third, reflective appreciation training did not simply cause confusion about preferences (Wilson and Schooler, 1991). If reflective appreciation training created confusion about preferences, then the liking of songs from the less preferred (favorite) subcategories should have shifted upward (downward) toward the mean, which did not occur.

## 6 Study 3

The prior studies provided evidence that encouraging reflective processing via appreciation training is effective for attitude change by increasing the liking of experiential goods from less preferred subcategories. Study 3 was designed to provide further evidence of this process by manipulating the type of thought participants had when listening to music. Participants were instructed to have evaluative thoughts (encouraging a reflexive response) or cognitive/affective thoughts (encouraging reflection). Appreciation should increase for a song from a less preferred subcategory when people are engaging in reflective thought (i.e., cognitive/affective thoughts) but not reflexive thought (i.e., evaluative thought).

### 6.1 Method

#### 6.1.1 Design and participants

Five hundred seventy-nine MTurk and Prolific Academic workers (median age = 35, 50.2% female workers, median education = bachelor's degree) participated in return for a payment. A power analysis determined we could detect an effect size of Cohen's *f* = 0.09 with 0.80 power. The study's design, hypotheses, planned sample size, inclusion/exclusion criteria, and planned primary analyses were preregistered (https://aspredicted.org/ZV2_3F1), and in accordance with the preregistration, participants were recruited in small batches across several days, different platforms, and accounts to ensure participants were not sharing the details of the study on blogs or forums. The only deviation from the preregistration was that all data were collected after the study preregistration. Although some data were collected before the preregistration, these data were discarded because of a programming error. There was no difference in participants recruited on either platform (*F* < 1), so the results were combined.

Participants were randomly assigned to one of four conditions in a 2 (training subcategory: favorite, less preferred) by 2 (response: reflexive, reflective) between-subject design. All participants received the appreciation training, and the dependent variable was liking of a novel target song in their third-ranked style. Eleven participants did not respond to the dependent variables, and 17 were removed from the analysis for failing to take the task seriously (“How seriously did you take this task?”; 6 or lower on a 9-point scale) in accordance with the preregistration, leaving a final sample of 551 people.

#### 6.1.2 Procedure

Similar to study 2, participants ranked the four styles of music that were illustrated using short song clips. Then, participants ranked their certainty about their ratings (1 = very uncertain, 7 = very certain). Consistent with the preregistered design, 11 participants who rated their certainty a three or lower, indicating they were uncertain about their music preferences, did not answer the dependent variables. As the procedure depends on music preferences, high uncertainty meant that we could not guarantee an accurate manipulation (i.e., if people do not have a favorite music style, it is hard to train them using their favorite style).

Next, all participants read that they were going to receive training to help them appreciate music. The training materials were the same as in the prior studies and focused on music appreciation, not factual information. Participants in the favorite training condition were trained on the tempo, melody, and texture using a sample song from their favorite subcategory. Participants in the less preferred training condition were trained on tempo, melody, and texture using a sample song from each of their three less preferred subcategories (as in study 2).

After completing the training, participants were told that they were going to listen to 80 s of a new song. This song was from their third-ranked style, consistent with study 1. Listening to this song was interrupted four times to encourage reflexive or reflective processing. Each interruption was structured as follows. First, participants were told they would have to create a sentence with a word. Then, participants were given a list of words to choose from. In the reflexive response condition, the list was composed of 10 evaluative words (i.e., like, average, enjoy, good, appreciate, excellent, bad, dislike, awful, and despise). In the reflective response condition, the list was composed of 10 cognitive words (i.e., beat, tempo, texture, melody, tune, rhythm, pitch, chorus, vocals, and instrumentals) and 10 affective words (i.e., happy, excitement, nostalgia, romantic love, calmness, sad, anger, pain, anxiety, and joy), randomly ordered.

After each interruption, participants chose one word. Then, participants were instructed to, “Please write a sentence about the song you were listening to. Please use [selected word] in the sentence.” After writing their sentence, participants repeated this process three additional times for a total of four 20-s clips with four-word selections and four sentences. Then, participants were asked to rate how much they liked, enjoyed, and appreciated the song from their third-ranked subcategory (1 = not at all, 9 = an extreme amount; α = 0.97). Afterward, they answered questions about their expertise, the credibility of the training, intrinsic motivation, and demographics.

### 6.2 Results

#### 6.2.1 Liking analysis

**Figure 4:**
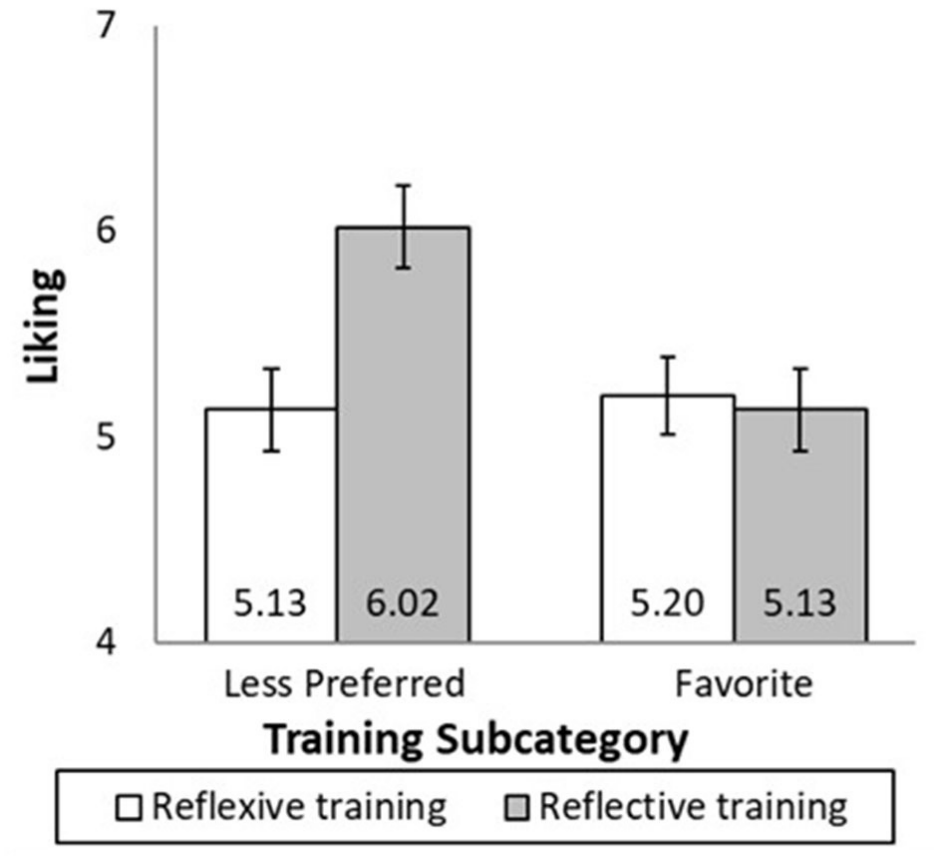
Liking of song from third-ranked subcategory. Participants received reflexive training or reflective training (between-subject factor) on songs from a less preferred or favorite subcategory (between-subject factor). Participants rated a novel song from their third-ranked subcategory. Reflective training only increases the appreciation of the song when training was in the less preferred subcategory. Error bars are ±1 SE.

The means are shown in Figure 4. Liking was subjected to a two-way analysis of variance (ANOVA) with the training subcategory and response as independent variables and liking as the dependent variable. There was a significant effect of the training subcategory [*F*_(1, 547)_ = 4.20, *p* = 0.041, *f* = 0.08] and response [*F*_(1, 547)_ = 4.32, *p* = 0.038, *f* = 0.08]. As predicted, there was a significant interaction between the training subcategory and response [*F*_(1, 547)_ = 5.93, *p* = 0.015, *f* = 0.09]. When people were trained using songs from their less preferred styles, encouraging reflective responses (*M* = 6.02, SD = 1.87) resulted in greater liking of the less preferred song than encouraging reflexive responses [*M* = 5.13, SD = 2.45; *F*_(1, 547)_ = 9.89, *p* = 0.002, *f* = 0.13]. When people were trained using the song from their favorite style, encouraging reflective responses (*M* = 5.13, SD = 2.31) and reflexive responses (*M* = 5.20, SD = 2.55) resulted in similar liking [*F*_(1, 547)_ = 0.07, *p* = 0.798].

#### 6.2.2 Alternative explanation

In the procedure, participants made comments on the songs using evaluative (reflexive) or cognitive/affective (reflective) words, which were the same words used in study 1. Yet, it is possible that the proportion of the negative words in the reflexive process list (40%) and the reflective process list (20%) was responsible for the effects on liking. In the reflexive response condition, the list was composed of six positive and four negative evaluative words (i.e., like, average, enjoy, good, appreciate, excellent, bad, dislike, awful, and despise). In the reflective response condition, the list was composed of 10 neutral cognitive words (i.e., beat, tempo, texture, melody, tune, rhythm, pitch, chorus, vocals, and instrumentals) and six positive and four negative affective words (i.e., happy, excitement, nostalgia, romantic love, calmness, sad, anger, pain, anxiety, and joy).

For example, participants may have used fewer negative words in the reflective—less preferred condition than in the reflective—favorite condition. The data showed only a main effect of processing type, with more negative words selected in the reflexive training condition (*M* = 0.38, SD = 0.67) than the reflective training condition [*M* = 0, SD = 0; *F*_(1, 547)_ = 90.72, *p* < 0.001, *f* = 0.40]. The fact that no negative words were used in either reflective condition cannot account for reflective training being more effective when the songs were from less preferred subcategories. For completeness, the interaction was not significant [*F*_(1, 547)_ = 0.51, *p* = 0.475].

### 6.3 Discussion

Study 3 manipulated the proposed process and demonstrated that appreciation increases for experiential goods from a less preferred subcategory but only when people engage in reflective appraisals that can modify or replace their automatic, reflexive responses. Those encouraged to only have reflexive responses showed less appreciation for experiential goods from a less preferred subcategory. Thus, it is only when people are engaging in cognitive or affective thought that changes or replaces their initial, reflexive perceptions are they able to revise their negative attitude and improve their appreciation for experiential goods from less preferred subcategories. This study provides evidence that manipulating the types of thoughts people have about experiential goods in the less preferred subcategories affects liking. Importantly, this only occurs when participants are trained on their less preferred experiential subcategories, which provides a greater opportunity for them to reevaluate their initial reflexive response.

## 7 Study 4

Study 4 provides evidence that reflection, and the resultant improvement to the initial, negative attitude toward less preferred subcategories, alters behavior (Krosnick and Petty, 1995). Thus far, people have undergone appreciation training and then been required to consume experiential goods in less preferred subcategories. If appreciation training is altering a person's ability to appreciate less preferred subcategories of experiential goods, then a person should be more willing to consume these goods when given a choice. We assessed this willingness by measuring the liking of the consumption experience, the intent to consume, and the amount of consumption.

Study 4 used visual art as a context for the study to further generalize the results to other experiential domains. The procedure used training framed as reflexive or reflective. Similar to study 3, the training used exemplars from a person's favorite or less preferred subcategories. Participants then responded to novel art in their favorite and less preferred subcategories. The study was conducted using three dependent measures: change in liking (study 4a), the intent to consume (study 4b), and the amount of consumption as indicated by time spent viewing art (study 4c).

### 7.1 Method

#### 7.1.1 Design

Participants were randomly assigned to one of four conditions in a 2 (training subcategory: favorite, less preferred) by 2 (training frame: reflexive conventional, reflective unconventional) between-subject design. Change in liking (4a), intention to consume (4b), and the amount of consumption (4c) were measured in three separate studies.

#### 7.1.2 Framing manipulation

The framing manipulation was designed to encourage reflexive (conventional training) vs. reflective (unconventional training) responses to the training and subsequent test material. The training frame was developed using considerable pretesting. The description of the reflexive training source was “The marketing team from a popular art reproduction company has developed the training program you are about to go through. The training will help you appreciate art.” We chose this as the source of the training in the reflexive training condition because we believed participants would view a marketing team for a company as espousing standard, established, and traditional ideas, which would encourage more reflexive responses to the artwork. The description of the reflective training source was “An art professor has developed the training program you are about to go through. The training will help you appreciate art.” We chose an art professor as the source of the training in the reflective training condition because we believed participants would view a university professor as teaching novel, unique, and alternate ideas, which would encourage more reflective appraisals of the artwork.

A manipulation check of these frames exposed a separate sample of 80 MTurk participants to the training frames. Participants then reported the degree to which they expected the training would encourage reflection, “make me spend more time thinking about the artwork,” “make me reflect more on the artwork,” “encourage me to think more deeply about the artwork,” “make me spend less time thinking about the artwork,” “make me make a snap judgment about the artwork,” and “encourage me to think superficially about the artwork” (the latter three items were reverse-coded; α = 0.80). The results showed a significant main effect of training frame [*M*_reflective_ = 5.38, SD = 1.10, *M*_reflexive_ = 4.12, SD = 1.14; *t*_(78)_ = 5.02, *p* < 0.001, *d* = 1.12], confirming that the reflective (reflexive) frame encouraged reflective (reflexive) thoughts.

#### 7.1.3 Procedure

The procedure of studies 4a−4c was largely the same up to the point of the dependent measures. First, participants ranked five styles of art: cubism, impressionism, neo-classicism, pop art, and realism. To assist participants in this process, each style was illustrated using an example painting. Participants were reminded to rank the entire style of art, not the example painting.

Next, participants received training on how to better appreciate art. The training was framed as reflexive or reflective. The training covered four topics: visual appeal, subject matter, meaning, and style. Training in each of the four areas involved three activities: (1) reading instructional information, (2) trying to apply the instructional technique to an accompanying painting, and (3) reading about an application of the technique to the same painting (see Supplementary material). For example, training on visual appeal in realism (1) told participants that:

Paintings should be visually appealing. This is best illustrated by Realism. Realist paintings are not always beautiful, but they do grab your eye. To illustrate, consider the Realist painting below. The artist uses the subject matter, color, and its realistic appearance to capture your attention. Please take a moment to examine the painting and reflect upon why it is visually appealing.

(2) asked participants, “Can you provide a reason the painting is visually appealing? Do not worry about coming up with a ‘correct' response. Appreciation is developed by trying.” and (3) discussed the specifics that contributed to the visual appeal of the accompanying painting (e.g., the contrast between warm and cool colors). This was similar to the procedure for the music appreciation training and focused on art appreciation, not factual information.

Participants in the favorite (less preferred) training condition were trained using four unique paintings from their favorite subcategory (one unique painting from each of their four less preferred subcategories). Thus, participants received the same instruction, but the source of the training encouraged either reflexive responses or reflective appraisals. The Supplementary material illustrate training in the less preferred condition for a person whose favorite subcategory is cubism and training in the favorite condition for a person whose favorite subcategory is realism.

##### 7.1.3.1 Study 4a dependent measure: change in liking

Participants viewed and rated 15 target paintings, three in each subcategory. Each painting was shown separately. Below the painting, participants were asked to rate their level of agreement with the statement, “I appreciate this painting for its …” (beauty, meaning, style, significance: 1 = completely disagree, 7 = completely agree) (scale adapted from Hager et al., 2012). Each painting was rated twice. The first rating was at the beginning of the procedure, immediately after ranking the five styles of paintings. The second rating was after the appreciation training was completed. Upon completion of the second rating, participants answered screening and demographic questions.

##### 7.1.3.2 Study 4b dependent measure: intention to view

After completing the training, participants imagined that they were visiting a museum with five rooms, each room showing a different style of art. They then rated their intention to view rooms with cubism, impressionism, neo-classicism, pop art, and realism (“How willing are you to enter each room?”; 1 = not at all willing, 7 = extremely willing). Afterward, they answered screening and demographic questions.

##### 7.1.3.3 Study 4c dependent measure: amount of consumption (view time)

After completing the training, participants viewed 15 target paintings in a simulated online museum. The 15 paintings consisted of three paintings from the five style subcategories. Each painting was shown separately. Participants were asked to view the paintings as naturally as possible, spending as much time as they wished with each painting. The paintings were randomly presented, and the time participants spent viewing each painting was surreptitiously recorded. After viewing the paintings, they answered screening and demographic questions.

### 7.2 Results

#### 7.2.1 Study 4a results: change in liking

##### 7.2.1.1 Sample

Two hundred nine MTurk workers (median age = 37, 53.6% female workers, median education = some college) participated in return for payment. Four participants failed to complete the training, leaving a final sample of 205 people. A sensitivity analysis determined we could detect an effect size of Cohen's *f* = 0.19 with 0.80 power.

##### 7.2.1.2 Changes in the liking of paintings from less preferred subcategories

The pre-training appreciation score was subtracted from the post-training appreciation score to calculate the change in liking. The liking scores for the three paintings in each of the four less preferred subcategories were averaged. The three-way interaction between the training subcategory, training frame, and the repeated-measure style factor (i.e., four less preferred subcategories) was not significant [*F*_(3, 603)_ = 0.96, *p* = 0.412], so the appreciation scores were averaged across the four less preferred subcategories.

**Figure 5:**
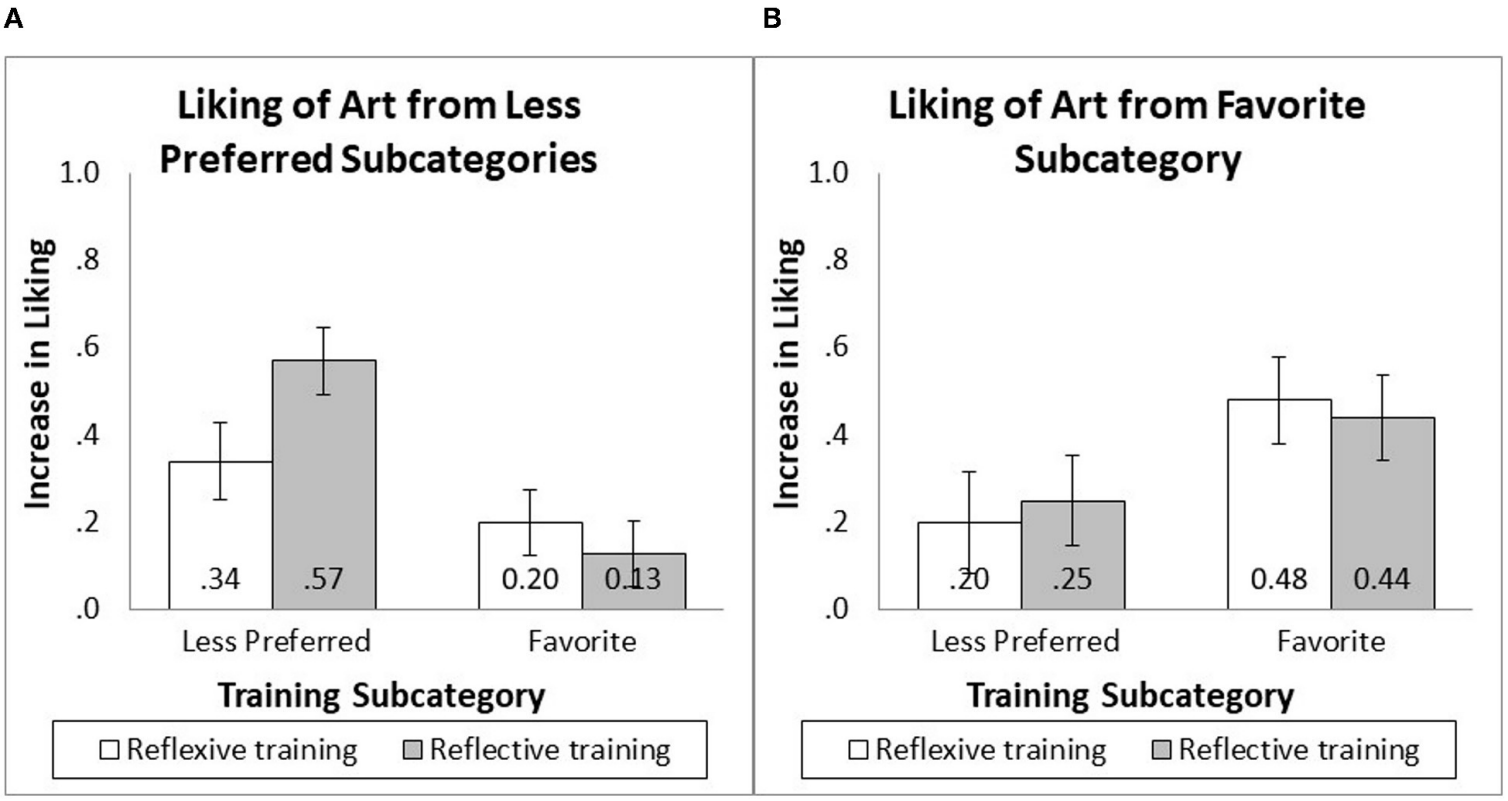
Changes in liking after appreciation training.

There was a significant effect of the training subcategory [*F*_(1, 201)_ = 12.85, *p* < 0.001, *f* = 0.24], an insignificant effect of training frame [*F*_(1, 201)_ = 1.05, *p* = 0.306], and a marginally significant effect of the predicted two-way interaction between the training subcategory and training frame [*F*_(1, 201)_ = 3.49, *p* = 0.063, *f* = 0.11]. When people were trained on their less preferred styles, reflective training (*M* = 0.57, SD = 0.58) resulted in a greater increase in liking of the paintings than reflexive training [*M* = 0.34, SD = 0.45; *F*_(1, 201)_ = 3.79, *p* = 0.053, *f* = 0.12]. When people were trained on their favorite style, reflective (*M* = 0.13, SD = 0.52) and reflexive (*M* = 0.20, SD = 0.66) training resulted in similar increases in liking [*F*_(1, 201)_ = 0.39, *p* = 0.531; see Figure 5A].

##### 7.2.1.3 Changes in the liking of paintings from the favorite subcategory

There was a significant effect of the training subcategory [*F*_(1, 201)_ = 4.95, *p* = 0.027, *f* = 0.14] such that training on favorite paintings (*M* = 0.46, SD = 0.77) improved the appreciation of paintings in favorite subcategories more than training in less preferred subcategories (*M* = 0.23, SD = 0.69). There was no significant effect of training frame [*F*_(1, 201)_ = 0.01, *p* = 0.940], or interaction between the training subcategory and training frame [*F*_(1, 201)_ = 0.17, *p* = 0.677], on appreciation for paintings from the favorite subcategory (see Figure 5B).

#### 7.2.2 Study 4b results: intention to view

##### 7.2.2.1 Sample

Two hundred sixty-three MTurk workers (median age = 31, 46.4% female workers, median education = some college) participated in return for a payment. Nineteen participants were removed for failing to properly complete the training, leaving a final sample of 244 people. A sensitivity analysis determined we could detect an effect size of Cohen's *f* = 0.17 with 0.80 power.

##### 7.2.2.2 Intention to view paintings from less preferred subcategories

**Figure 6:**
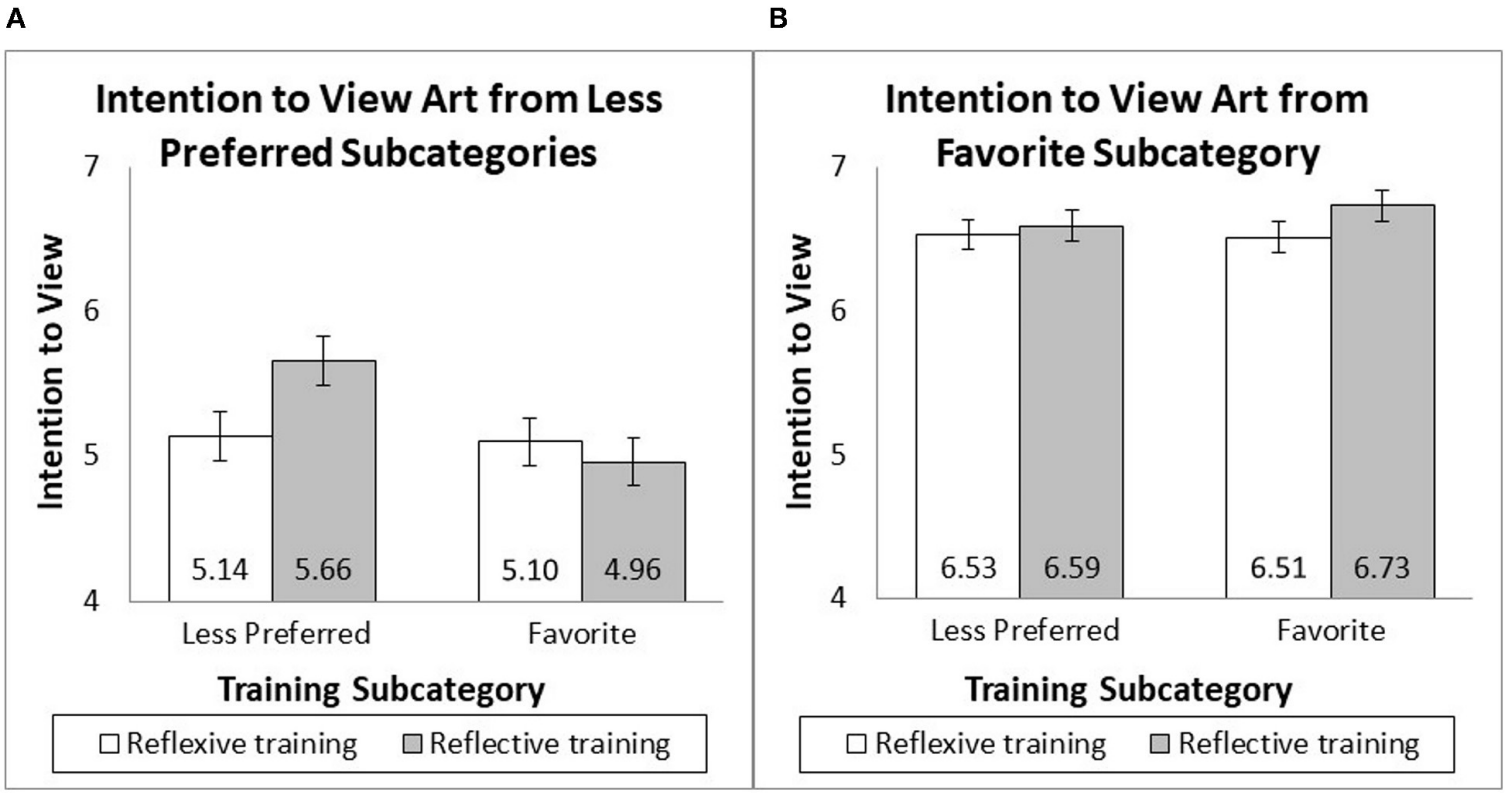
Intention to view art after appreciation training.

There was no interaction of the four less preferred subcategories with the training frame [*F*_(4, 224)_ = 2.05, *p* = 0.088], training subcategory [*F*_(4, 224)_ = 0.57, *p* = 0.685], or the interaction of the two [*F*_(4, 224)_ = 1.12, *p* = 0.348], so participants' intention to view their four less preferred painting styles was averaged. The intention to view ratings was subjected to a two-way analysis of variance (ANOVA) with the training subcategory and training frame as independent variables. There was a significant effect of the training subcategory [*F*_(1, 240)_ = 4.91, *p* = 0.028, *f* = 0.13] but no significant effect of training frame [*F*_(1, 240)_ = 1.28, *p* = 0.260]. As predicted, there was a significant interaction between the training subcategory and training frame [*F*_(1, 240)_ = 3.94, *p* = 0.048, *f* = 0.11]. When people were trained using paintings from their less preferred styles, reflective training (*M* = 5.66, SD = 1.09) resulted in more intention to view the paintings from less preferred styles than reflexive training [*M* = 5.14, SD = 1.36; *F*_(1, 240)_ = 4.81, *p* = 0.029, *f* = 0.13]. When people were trained using paintings from their favorite style, reflective (*M* = 4.96, SD = 1.20) and reflexive (*M* = 5.10, SD = 1.52) training resulted in a similar intention to view paintings from less preferred styles [*F*_(1, 240)_ = 0.37, *p* = 0.544 see Figure 6A].

##### 7.2.2.3 Intention to view paintings from the favorite subcategory

There was no significant effect of the training subcategory [*F*_(1, 240)_ = 0.26, *p* = 0.608], training frame [*F*_(1, 240)_ = 1.74, *p* = 0.189], or an interaction between the training subcategory and training frame [*F*_(1, 240)_ = 0.55, *p* = 0.459] on the intention to view paintings from the favorite subcategory (see Figure 6B).

#### 7.2.3 Study 4c results: amount of consumption (view time)

##### 7.2.3.1 Sample

Two hundred five MTurk workers (median age = 32, 56.6% female workers, median education = some college) participated in return for a payment. One participant was removed for failing to complete the study, leaving 204 people for analysis. A sensitivity analysis determined we could detect an effect size of Cohen's *f* = 0.20 with 0.80 power.

##### 7.2.3.2 Data preparation

Contingent on the participants' favorite painting style, the time spent on each painting in the four less preferred painting styles (12 paintings) was summed. An initial analysis indicated there were extreme values. For example, the aggregate viewing time showed a mean of 122.24, a standard deviation of 225.36, and a range of 4.35 to 1,206.63 s. Given the propensity of extreme values to bias statistical tests, the extreme values were truncated (McClelland, 2000). We used the median absolute deviation method (MAD) to determine the extreme values and truncation values (Leys et al., 2013). The MAD method does not rely on the mean or standard deviation to identify cutoffs for extreme values. Hence, it is not subject to the criticism that outliers are influencing the criteria that determine their classification, as is the case with the deviation from the mean method.

The MAD method (a) determines the median, (2) assesses absolute deviations from the median, (c) determines the median of these absolute deviations, and (d) determines a threshold deviation from the median, after adjusting for normality. Leys et al. (2013) recommend a threshold value of 2.5 median deviations, implying a cutoff that should capture 98.8% of the distribution. Applying this method to the viewing time data, 14 observations above 193.39 s were truncated to this value. The lower bound included zero, so there were no truncations of short viewing times.

##### 7.2.3.3 Viewing time of paintings from less preferred subcategories

**Figure 7:**
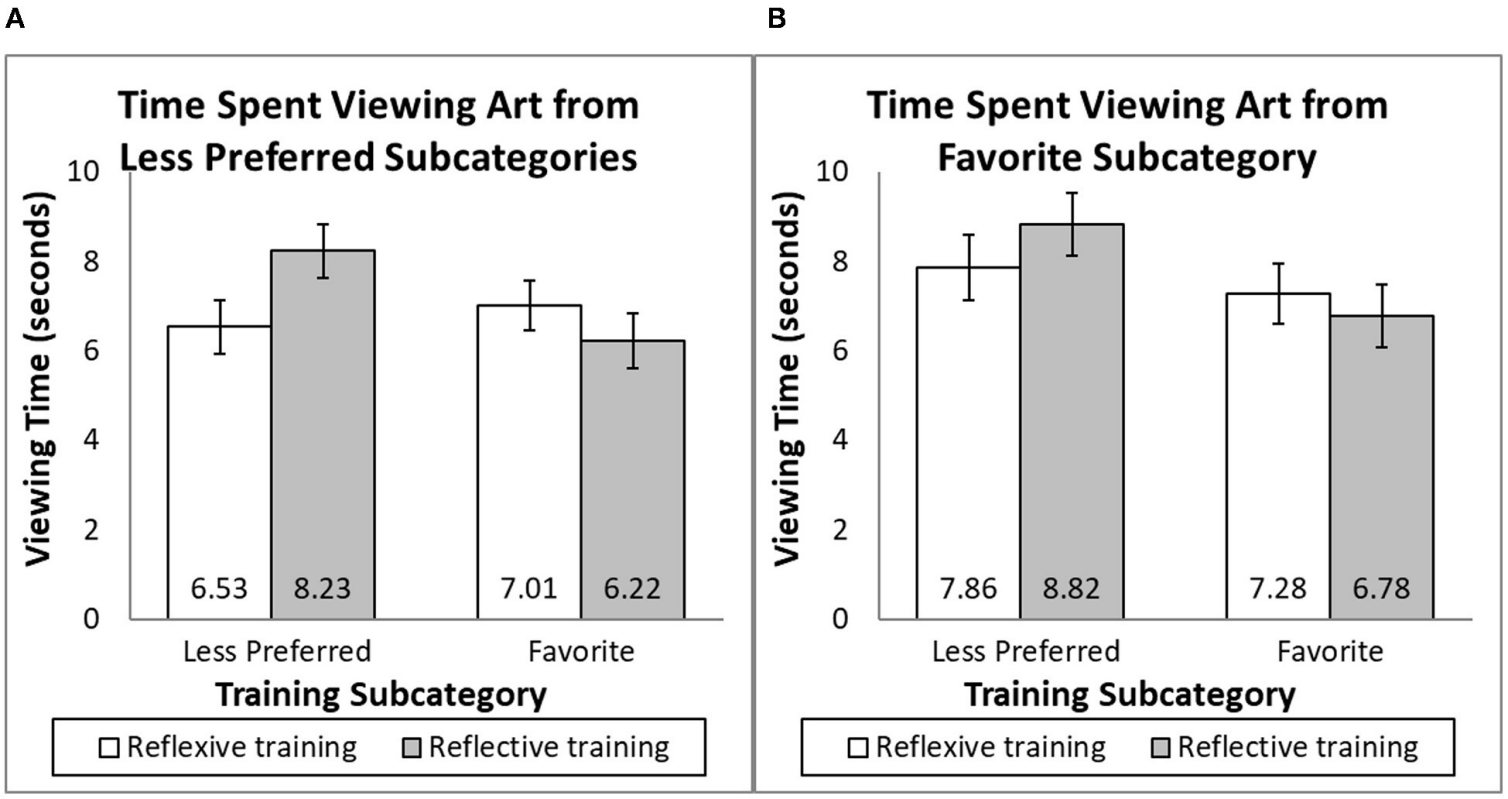
Amount of consumption (view time) after appreciation training.

There was no interaction of the four less preferred subcategories with the training frame [*F*_(4, 184)_ = 0.35, *p* = 0.844], training subcategory [*F*_(4, 184)_ = 1.23, *p* = 0.300], or the interaction of the two [*F*_(4, 184)_ = 0.71, *p* = 0.586], so participants' viewing time of their four less preferred painting styles was averaged. The view time data were subjected to a two-way analysis of variance (ANOVA) with the training subcategory and training frame as independent variables and viewing time (in seconds) as the dependent variable. There was no significant effect of the training subcategory [*F*_(1, 200)_ = 0.59, *p* = 0.442] or training frame [*F*_(1, 200)_ = 1.68, *p* = 0.196]. As predicted, there was a significant interaction between the training subcategory and training frame [*F*_(1, 200)_ = 4.44, *p* = 0.036, *f* = 0.13]. When people were trained using paintings from their less preferred styles, reflective training (*M* = 8.23, SD = 4.47) resulted in more viewing of the paintings than reflexive training [*M* = 6.53, SD = 3.80; *F*_(1, 200)_ = 4.03, *p* = 0.046, *f* = 0.12]. When people were trained using paintings from their favorite style, reflective (*M* = 6.22, SD = 4.19) and reflexive (*M* = 7.01, SD = 4.34) training resulted in similar viewing times [*F*_(1, 200)_ = 0.92, *p* = 0.339; see Figure 7A].

##### 7.2.3.4 Intention to view paintings from the favorite subcategory

The viewing times for the three paintings from the favorite subcategory were averaged, MAD truncated, and analyzed. There was a marginally significant effect of the training subcategory [*F*_(1, 200)_ = 3.50, *p* = 0.063, *f* = 0.11], a non-significant training frame [*F*_(1, 200)_ = 0.11, *p* = 0.744], and a non-significant training subcategory by training frame interaction [*F*_(1, 200)_ = 1.10, *p* = 0.296] on the time spent viewing paintings from the favorite subcategory (see Figure 7B).

### 7.3 Discussion

Studies 4a, 4b, and 4c found that reflective appreciation training in less preferred subcategories changed the attitude toward the art by increasing liking, increased the intent to view other art in those subcategories, and prolonged the amount of time spent viewing the art. This suggests that the appreciation training encouraging reflection altered the way the sensory information of that experience was assessed, so that new experiential goods in less preferred subcategories could be better appreciated, which impacted subsequent consumption behavior. Importantly, appreciation for art in the favorite subcategory did not change. Reflexive vs. reflective training on art from the favorite subcategory did not impact the change in liking, intent to view, or viewing time of art in the favorite subcategory.

Importantly, the evidence from study 4b does not support the conclusion that mere exposure to paintings from less preferred subcategories improves interest and appreciation. The responses to paintings from less preferred subcategories were assessed using novel paintings, so there was no repeated exposure. Moreover, if mere exposure was creating familiarity with elements (not exemplars) of a style, there should have been a main effect of the training subcategory, not the observed interaction. We also note that variety-seeking behavior cannot explain the results of study 4c. If people were seeking variety, then there should have been a main effect of the training subcategory, wherein training with favorite art (vs. less preferred art) led to more subsequent viewing of paintings from a less preferred subcategory.

## 8 General discussion

The results of six studies demonstrate that people's negative attitudes can change as they learn to appreciate experiential goods in less preferred subcategories. The studies show that appreciation training encouraging reflection during a consumption experience allows people to override more reflexive responses to experiential goods in less preferred subcategories (study 1). Reflective appreciation training encourages positive cognitive and affective responses to new experiential goods in less preferred subcategories (study 2). Reflective appreciation training must occur on experiential goods from less preferred subcategories, so that new associations can generalize to novel experiential goods in less preferred subcategories (all studies).

### 8.1 Future research

Reflection is a strategy that relies on reappraisal but also incorporates learning. Reappraisal typically involves detachment (i.e., ignoring negative appraisals from an experience) (Shiota and Levenson, 2009) and positive reappraisal (i.e., focusing on positive appraisals from an experience) (Shiota, 2006). These strategies are used to alter affective responses (e.g., sadness, anger, and fear) to an event (Ochsner and Gross, 2005) as well as cognitive responses (Carbone and Haeckel, 1994). Reflection relies on these strategies but goes one step further by encouraging people to link specific sensory information to positive appraisals. The learning is subcategory-specific, in that reflection using a less preferred subcategory enhances the appreciation of experiences with other experiential goods in that same subcategory. The research issue is how to isolate factors that establish, reinforce, and generalize the sensory cue–positive appraisal associations that develop during reflection. Future research could investigate how the intensity of the positive appraisal (i.e., conditioning theory), the frequency of the sensory cue–positive appraisal pairing (i.e., operant conditioning theory), the number of sensory cues that link to the positive appraisal (i.e., schema theory), and the accessibility of these associations contribute to the change in appreciation for consumption experiences from the less preferred subcategory.

A second research opportunity is to determine how sensitive reflection is to the characteristics of the experiential goods (e.g., music, art, film, wine, and food). These domains can vary on several important dimensions, including sensory complexity (e.g., music involves one sensory system whereas a movie involves multiple), the degree of cognitive mediation (e.g., movie consumption is more cognitively mediated than food consumption), and the difficulty of synthesizing sensory information into appreciation (e.g., it is easier to “learn” how to appreciate food than art) (Spence, 2011). It is important to examine the degree to which sensory complexity, opportunities for cognitive mediation, and the (non)orthogonality of positive appraisals inhibit or facilitate how sensory cues evolve into positive appraisal learning and generalization.

A third research opportunity involves developing a better understanding of how the interplay between the reflexive response and reflective appraisals influences attempts to alter how people experience negative events. We have assumed a serial relationship between the reflexive and reflective processes (i.e., reflexive responses happen before reflective responses) (Kahneman and Frederick, 2002). The implication is that the reflexive response and reflective appraisal can rely on the same sensory information of the consumption experience. An alternative assumption is a parallel relationship between the reflexive and reflective processes (Evans and Stanovich, 2013). The implication is that the reflexive response and reflective appraisals can rely on different sensory characteristics of the consumption experience. It is likely that each type of model applies to a subset of consumption experiences. Unlike the experiential goods (music, art, film, wine, or food) focused on in this study, we hypothesize that consumption experiences that generate more physiological (e.g., allergies and pain), visceral (e.g., fear and disgust), or phobic (e.g., acrophobia and social phobias) responses are more likely to encourage parallel processing, as opposed to serial processing, because the sensory cues are intense. In these cases, more reflection will not alter the negative responses. Desensitization may be the best strategy for changing negative attitudes to these types of experiences.

A fourth avenue for future research is understanding how to reinforce the new associations generated by reflective appraisals. Associations generated by reflective appraisal compete with associations generated by reflexive responses. The opportunity is to investigate ways to encourage the initiation and reinforcement of reflective appraisals so that they become reflexive. Possibilities include making the sensory cues that support the reflective appraisal more vivid (Blondé and Girandola, 2016), more expected (Summerfield and de Lange, 2014), and more perceptually relevant (Goldstone, 1994). Each of these strategies has been investigated in other domains but not with respect to redefining the value of experiential goods.

Finally, future research could examine the extent to which appreciation training improves overall wellbeing. Research by Oishi and Westgate (2022) demonstrates that there are three dimensions of the good life: a happy life, a meaningful life, and a psychologically rich life. Psychological richness occurs when one has a variety of interesting and perspective-changing experiences, both of which characterize our appreciation training, namely, our appreciation training should result in perspective changes for less preferred experiential subcategories which would increase the variety of subsequent consumption experiences (i.e., encouraging attitude change should result in people being open to considering and consuming a wider variety of experiential goods). Without psychological richness, there is the risk that experiences become “monotonous, dreadful, and boring”. Thus, future research should explore the relationship between appreciation training, psychological richness, and overall wellbeing.

### 8.2 Limitations

Our investigation into how to encourage attitude change by increasing the appreciation of experiential goods from less preferred subcategories has several important limitations. First, it does not identify the specific associations that were created during reflection. Perceptions of the sensory characteristics of an experience are idiosyncratic and personal (Marcel, 1983). Cognitive and affective responses to an experience are also idiosyncratic and personal (Holbrook and Hirschman, 1982; Cohen and Areni, 1991). Thus, we can provide evidence for their existence (studies 1 and 2), what was chosen from a predetermined list (study 3), and the impact (studies 1–4) of associations created during reflection, but not their composition.

Second, we do not investigate the conditions under which appreciation training frames (studies 1, 2, and 4) and type of thinking (study 3) encourage reflection and, consequently, new, beneficial associations. Reflection is typically an endogenously motivated activity. It is motivated by curiosity and the importance of an event to the self (Arnold, 1960a,b; Kappas, 2006). Making the motivation for reflection exogenous, as with our studies, necessitates an intervention. This intervention can be initiated by the person who delivers the experience (e.g., a musician introducing her work), the retailer who promotes the experience (e.g., a waiter explaining the impact of the ingredients in an entrée), or the guide who manages the experience (e.g., a museum docent). We can only speculate on the moderators of these exogenous motivation techniques. For example, it may be that an experiential category must be important (Kalisch et al., 2015), the consumer must have sufficient consumption knowledge (Clarkson et al., 2013), and/or the consumer must be motivated to enjoy the experience (McRae et al., 2012) for reflection to be beneficial.

Finally, appreciation training is more likely to be successful when the consumption experiences of subcategories are more homogeneous than heterogeneous. To illustrate, consider the product category of alcohol and the subcategories beer, wine, hard alcohol, and liqueurs vs. the product category white wine and the subcategories pinot grigio, chardonnay, riesling, and sauvignon blanc. We anticipate that the diversity of the consumption experiences in the alcohol subcategories would make it difficult for appreciation training to have an impact in less preferred subcategories—the cross-product experiences are too diverse for the experiences in the most preferred category to enable the benefits of reflection in less preferred subcategories. It is only when there is some overlap in the experiences of subcategories (e.g., white wine subcategories) that there is sufficient knowledge to enable the benefits of reflection in a less preferred subcategory. Said another way, a person's experiential knowledge base must be sufficient to make reflection beneficial.

## Data availability statement

The datasets, analysis code, and research materials are available in the OSF repository: https://osf.io/e8wh4/?view_only=36ddc730e9bb4dd084ee0b8b003c29b1.

## Ethics statement

The studies involving humans were approved by University of Florida UFIRB 2011-U-931 and University of Oxford CUREC_1A SSH_SBS_C1A_16_068. The studies were conducted in accordance with the local legislation and institutional requirements. The participants provided their written informed consent to participate in this study.

## Author contributions

CC: Conceptualization, Data curation, Formal analysis, Writing—original draft, Writing—review & editing. CJ: Conceptualization, Formal analysis, Supervision, Writing—original draft, Writing—review & editing.
