# Supplementary material for: Cognitive and affective reflection increases appreciation for less preferred subcategories of experiential goods
Source: Front Psychol. 2023 Dec 21;14:1271516. doi: 10.3389/fpsyg.2023.1271516 (PMC10764625; doi:10.3389/fpsyg.2023.1271516)
Supplement: Supplementary file 1 [file Data_Sheet_1.docx]

Supplementary Material

Please see OSF repository for painting images: https://osf.io/e8wh4/?view_only=36ddc730e9bb4dd084ee0b8b003c29b1

# Study 4 Art Training Examples

| **Training Using Less-Preferred Subcategories for People Whose Favorite Subcategory is Cubism** | | | |
| --- | --- | --- | --- |
|  | **Instructional Information** | **Application** | **Application Feedback** |
| Step 1: Visual Appeal  Realism | Paintings should be visually appealing. This is best illustrated by Realism. Realist paintings are not always beautiful, but they do grab your eye. To illustrate, consider the Realist painting below. The artist uses the subject matter, color, and its realistic appearance to capture your attention. Please take a moment to examine the painting and reflect upon why it is visually appealing.  <<similar to Brass Bowl and Blood Oranges by Dorothea Schulz>> | Can you provide a reason the painting is visually appealing? Do not worry about coming up with a "correct" response. Appreciation is developed by trying. | For example, the Realism painting below appeals to the eye because of the vibrant contrast between the warm colors (oranges) and the cool colors (blues). The artist wants to draw your eye into the center of the painting. The angle of the sliced fruit, the use of color, and how the light shines the most brightly on the halved orange all invites your eye to one focal point. |
| Step 2: Subject Matter  Neo-Classicism | Ask yourself, “**What is the subject of the painting?**” For Neo-Classicism, the subject matter is often a famous person, mythical gods, an epic or biblical story, or a famous battle or city. The subject matter is carefully crafted to reference historical, biblical, or mythical people and events.  <<Aurora and Cephalus by Pierre-Narcisse Guérin>> | Can you tell us something about the subject matter? Again, do not worry about coming up with a "correct" response. | The subject of the painting is Aurora, goddess of dawn, and Cephalus, a mortal. The painting depicts the story of Aurora who falls in love with Cephalus and kidnaps him while he is hunting. She holds him captive in the sky and they are briefly lovers until Aurora releases him because Cephalus misses his wife. |
| Step 3: Meaning  Pop Art | Look for meaning in Pop Art paintings. For example, they often involve elements of cultural or political commentary or ironic or satirical elements. Paintings can include multiple meanings, so instead of trying to find THE meaning of a painting, **focus on what the painting says to you**.  <<Converse Shoes by Andy Warhol>> | Can you identify one meaning of the painting? | One meaning of Pop Art is to take elements of popular culture and elevate it to art-worthy, defying the elitist culture of most painting styles. Pop Art commonly uses irony to depict a consumer product or cultural icon in an overtly kitschy way. Even the painting process, screen printing, is a semi-mechanized way of putting paint on canvas and is typical of Pop Art, which reinforce the symbolism of mass production giving everyone access to art. |
| Step 4: Style  Impressionism | Impressionism artists follow a well-established style of painting almost haphazardly. **Style can also convey meaning.** For example, Impressionism often uses wide, quick strokes to convey motion and capture the mood and feel of the scene, purposely leaving out details.  <<Impression Sunrise by Claude Monet>> | Can you identify a style component and tell us what it means to you? | In this Impressionist painting, the artist uses the typical, loose painting style. Paint appears to be applied quickly and the brush strokes are still visible, instead of carefully blended together. The intent of this style is to show a quick impression of the harbor scene by capturing the feeling and colors, like the colors of the sky and sea at twilight and calm return of the small boats to safe moorings for the night. |

| **Training on Favorite Subcategory for People Whose Favorite Subcategory is Realism** | | | |
| --- | --- | --- | --- |
|  | **Instructional Information** | **Application** | **Standard Application** |
| Step 1: Visual Appeal  Realism | Paintings should be visually appealing. This is best illustrated by Realism. Realist paintings are not always beautiful, but they do grab your eye. To illustrate, consider the Realist painting below. The artist uses the subject matter, color, and its realistic appearance to capture your attention. Please take a moment to examine the painting and reflect upon why it is visually appealing.  <<similar to Brass Bowl and Blood Oranges by Dorothea Schulz>> | Can you provide a reason the painting is visually appealing? Do not worry about coming up with a "correct" response. Appreciation is developed by trying. | For example, the Realism painting below appeals to the eye because of the vibrant contrast between the warm colors (oranges) and the cool colors (blues). The artist wants to draw your eye into the center of the painting. The angle of the sliced fruit, the use of color, and how the light shines the most brightly on the halved orange all invites your eye to one focal point. |
| Step 2: Subject Matter  Realism | Ask yourself, “**What is the subject of the painting?**” For Realism, the subject matter is often a person, an animal, or a collection of everyday items. The subject matter is carefully crafted to reflect reality and there is usually a clearly defined object that is the subject of a Realism painting.  <<Bittersweet with Blue by Barbara Groff>> | Can you tell us something about the subject matter? Again, do not worry about coming up with a "correct" response. | The subject matter in the Realism painting below is of vases and fabric sitting on a table. This painting features a common collection of items typical of Realism paintings. It is an exercise on painting what you see, or how it is represented in reality. The colors are slightly enhanced beyond reality to be more beautiful and inviting. |
| Step 3: Meaning  Realism | Look for meaning in Realism paintings. For example, they often involve elements of simplicity or elegance. Paintings can include multiple meanings, so instead of trying to find THE meaning of a painting, **focus on what the painting says to you**.  <<similar to Dame Wendy Margaret Hiller by Thomas Cantrell Dugdale>> | Can you identify one meaning of the painting? | A major theme of the Realism painting is “beauty in simplicity.”  There is beauty in all of the normal things that surround us if we would only take the time to appreciate it, which is a common meaning in realism paintings. This “beauty in simplicity” theme is further reinforced by the unadorned background, plain clothing, and neutral colors. |
| Step 4: Style  Realism | Realism artists follow a well-established style of painting using controlled strokes while still maintaining elements of the brushstrokes. The painting is intended to look realistic without looking like a photo. **Style can also convey meaning.** For example, Realism often uses blending and small strokes to incorporate details but maintains elements that are not realistic, like exaggerated colors or a stylized background.  <<Orchid Fandango by Elizabeth Horning>> | Can you identify a style component and tell us what it means to you? | In this Realism painting, the artist uses the typical, painting style that follows the rules of perspective, lighting, and relationship. The painting does not look like a photograph and the elements of the paint and brush strokes are preserved. The use of a stylized depiction of the flowers allows the artist to use color and light to enhance the scene, and make a less attractive reality more beautiful and impressive. |

# Study 1-3 Music

## Sample Songs Used for Ranking and Training

| Style | Artist and Song Title | Song Link |
| --- | --- | --- |
| Classical | Vivaldi  The Four Seasons: Autumn | <https://w.soundcloud.com/player/?url=https%3A//api.soundcloud.com/tracks/1201257> |
| Hip Hop | Nelly featuring Kelly Rowland  Dilemma | <https://w.soundcloud.com/player/?url=https%3A//api.soundcloud.com/tracks/259821471> |
| Pop | Ed Sheeran  Perfect | <https://w.soundcloud.com/player/?url=https%3A//api.soundcloud.com/tracks/310108203> |
| Rock | Nirvana  Heart-Shaped Box | <https://w.soundcloud.com/player/?url=https%3A//api.soundcloud.com/tracks/258954379> |

## Songs Used for Dependent Variable

| Style | Artist and Song Title | Song Link |
| --- | --- | --- |
| Classical | Jennifer Higdon  Violin Concerto - Chaconni | https://w.soundcloud.com/player/?url=https%3A//api.soundcloud.com/tracks/341533499 |
| Hip Hop | Juice WRLD  Moonlight | https://w.soundcloud.com/player/?url=https%3A//api.soundcloud.com/tracks/328259792 |
| Pop | Evalyn  Angels | <https://w.soundcloud.com/player/?url=https%3A//api.soundcloud.com/tracks/473969427> |
| Rock | The Grove  World I Used to Know | <https://w.soundcloud.com/player/?url=https%3A//api.soundcloud.com/tracks/73091692> |

# Study 4a-4c Art

## Sample Artwork Used for Ranking and Less-Preferred Training

| Style | Artwork |
| --- | --- |
| Cubism | Girl Before a Mirror by Pablo Picasso |
| Impressionism | Impression Sunrise by Claude Monet |
| Neo-Classicism | Aurora and Cephalus by Pierre-Narcisse Guérin |
| Pop Art | Converse Shoes by Andy Warhol |
| Realism | similar to Brass Bowl and Blood Oranges by Dorothea Schulz |

## Sample Artwork Used for Favorite Training

| Style | Artwork |
| --- | --- |
| Cubism | Girl Before a Mirror by Pablo Picasso  Harlequin by Pablo Picasso  Portrait of Picasso 1936 by Dora Maar  Three Musicians by Pablo Picasso |
| Impressionism | Impression Sunrise by Claude Monet  Untitled by Ekaterina Zacharova  Northern Landscape by Glen W. Ferguson  Water Lilies by Claude Monet |
| Neo-Classicism | Aurora and Cephalus by Pierre-Narcisse Guérin  The Nightmare by Henry Fuseli  Napoleon I on His Imperial Throne by Jean Auguste Dominique Ingres  The Cupid Seller by Joseph-Marie Vien |
| Pop Art | Converse Shoes by Andy Warhol  Campbell’s Soup Cans by Andy Warhol  Green Coca-Cola Bottles by Andy Warhol  Triple Elvis by Andy Warhol |
| Realism | similar to Brass Bowl and Blood Oranges by Dorothea Schulz  Bittersweet with Blue by Barbara Groff  similar to Dame Wendy Margaret Hiller by Thomas Cantrell Dugdale  Orchid Fandango by Elizabeth Horning |

## Artwork Used for Dependent Variable Studies 4b-4c

| Style | Artwork |
| --- | --- |
| Cubism | All that Jazz by Kristen Stein  Composition with Figures by Liubov Popova  Le Pigeon aux Petits Pois by Pablo Picasso |
| Impressionism | similar to By the Evening by Alston Yuriy Lobachov  Acrylic Landscape by Will Kemp  similar to Lucky by Anita Mosher Solich |
| Neo-Classicism | La Jeune Dame en robe bleue by Adélaïde Labille-Guiard  Portrait of Princess Anna Gagarina and Princess Varvara Gagarina by Vladimir Borovikovsky  The Nags Head Portrait by Theodosia Burr |
| Pop Art | Marilyn Monroe by Stephane Piovan-Draw  Grevy’s Zibra by Pablo Picasso  Star Wars screen print by Eelus |
| Realism | Realist Oil Painting of a Pear by Daniel C. Chiriac  similar to Coconut Tree by Paula Marie Leslie  Team Leader by Vasili K. Nechitailo |
